# Survivability of *Clostridioides difficile* spores in fermented pork summer sausage during refrigerated storage

**DOI:** 10.14202/vetworld.2022.162-167

**Published:** 2022-01-27

**Authors:** Genevieve Flock, Hsin-Bai Yin, Chi-Hung Chen, Abraham Joseph Pellissery, Kumar Venkitanarayanan

**Affiliations:** 1 Combat Capabilities Development Command Soldier Center, Soldier Sustainment Directorate, Combat Feeding Division, Natick 01760, Massachusetts, United States; 2 Department of Agriculture, USDA Agricultural Research Service, Beltsville, Maryland 20705, United States; 3 Department of Animal Science, University of Connecticut, College of Agriculture Health and Natural Resources, Mansfield 06269, Connecticut, United States.

**Keywords:** acidity, *Clostridioides difficile*, fermented pork sausage, spores

## Abstract

**Background and Aim:**

*Clostridioides difficile* is a spore-forming pathogen that causes serious enteric disease in humans. Strains have been isolated from food animals and meat, including pork, which suggest a potential for foodborne transmission. Pork summer sausage is a popular fermented meat product, which is consumed cooked or cooked to a lower internal temperature due to acidification of the product. The effect of acidity and cooking on the viability of *C. difficile* spores in a fermented meat product has not been determined. Therefore, the aim was to study the survivability of *C. difficile* spores in fermented pork summer sausage.

**Materials and Methods:**

Fermented pork sausages were prepared according to a commercial recipe with or without starter culture and *C. difficile* spores followed by fermentation at 37°C for ~12 h under 85% relative humidity until pH 5.0 was reached and further processed as cooked (>57°C) or uncooked (≤57°C) and stored at 4°C. *C. difficile* spores in sausages were enumerated at 1 h following inoculation and on days 0, 1, 7, 14, 21, 30, 60, and 90 of storage.

**Results:**

It was observed that *C. difficile* spore viability in control unfermented treatment was significantly different on day 0 from the fermented, fermented cooked, and control unfermented cooked treatments (p<0.05); however, there was no significant difference among the latter three treatment groups throughout 90 days of storage (p>0.05). On day 90 of storage, the unfermented control sausages yielded ~4.0 log colony-forming unit (CFU)/g of *C. difficile* spores compared to ~3.5 log CFU/g recovered from fermented samples and the unfermented cooked control samples identifying spore viability in all treatment groups.

**Conclusion:**

*C. difficile* spores were found to survive the acidity and cooking of fermented pork summer sausage and storage at 4°C for 3 months, thereby highlighting the need for effective intervention strategies to reduce the risk of *C. difficile* contamination in pork products.

## Introduction

*Clostridioides difficile* is a Gram-positive, anaerobic nosocomial pathogen which causes a serious and potentially life-threatening toxin-mediated disease [1,2]. The infection is characterized by abdominal pain and diarrhea, leading to more serious clinical manifestations such as colitis and toxic megacolon [3]. Although traditionally regarded as a nosocomial infection, especially in those receiving antimicrobial therapy [4,5], a newer epidemiological trend observed in *C. difficile* incidence is an increase in the number and severity of infections in humans, particularly those involving communities outside the hospital environment [6,7]. Further, *C. difficile* infections have been increasingly diagnosed in low-risk individuals who are younger with no history of antibiotic treatment [8,9]. Many investigators have reported the occurrence of *C. difficile* in a variety of food animals, and a potential reason attributed to increased reports of human *C. difficile* infections is the rise in isolation rates of the pathogen from animal reservoirs [10,11]. *C. difficile* has been isolated from raw and ready-to-eat (RTE) meats such as pork, intended for human consumption at retail stores [12-14]. The detection of genotypically similar and identical *C. difficile* strains implicated from human infections in food animals and foods [15-17] further strengthens the potential role of food as a source of community-associated *C. difficile* infection [18,19]. However, additional research is recommended to understand the relationship and potential risk of *C. difficile* in foods and community-associated infections.

*C. difficile* is a major cause of enteritis in neonatal pigs [20,21], leading to significant mortality in suckling piglets and high isolation rates of up to 35% [22,23]. *C. difficile* has also been isolated from live adult pigs at rates up to 23%, with high colonization rates in sows around the time of farrowing [17,24]. With swine as a potential reservoir of *C. difficile*, contamination of swine carcasses at the time of slaughter could lead to downstream contamination of pork and other RTE pork products. In addition, a survey involving sausage processing plants in Texas and retail meat locations isolated *C. difficile* from 9.5% (23/243) of meat samples and swab samples collected over a 5-year period [25]. Additional prevalence studies in the US found that 62.5% of RTE pork braunschweiger and (3/15) braised skin and colon RTE pork products tested positive for *C. difficile* [26,27]. Thus, *C. difficile* isolation in live pigs, processing facilities, and retail pork products identifies the potential risk of *C. difficile* contamination in RTE pork products.

Pork summer sausage is a popular fermented meat product in the United States. It can be classified as cooked (internal temperature >57°C) or uncooked to a lower temperature (internal temperature ≤57°C), since acidification due to fermentation is believed to kill any surviving vegetative pathogens in the product [28,29]. In addition, the product is typically consumed without an additional cooking step. The starter culture, *Pediococcus acidilactici*, is commonly used in fermented sausage to produce lactic acid as a by-product of fermentation, which, in turn, lowers the product pH. If *C. difficile* spores could survive the pH and cooking temperature employed in fermented pork summer sausage, this RTE product could be a potential source of the pathogen.

Since no previous study investigated the viability of *C. difficile* spores in product in the study area, the objective was to determine the viability of *C. difficile* spores in RTE fermented pork summer sausage.

## Materials and Methods

### Ethical approval

Ethical approval was not required for this study because there was no involvement of animal or human subjects.

### Study period and location

The study was conducted from February to September 2015 at the University of Connecticut in Storrs, CT.

### Spore preparation

*C. difficile* spores were prepared using a previously published protocol with slight modifications [30]. Briefly, single colonies of *C. difficile* ATCC BAA 1805, 1803, and 1053 (human clinical isolates) were separately inoculated into brain heart infusion broth supplemented (BHIS) with 5% yeast extract (Oxoid, Hampshire, UK) and cultured overnight at 37°C under anaerobic conditions. A 150 mL aliquot of the overnight culture was gently spread to evenly distribute the culture onto BHIS agar (Oxoid) in 6-well plates and was cultured anaerobically for 7 days at 37°C in a Whitley A35 anaerobic workstation (Microbiology International, Frederick, Maryland, United States) to allow sporulation. After 7 days, spores were harvested from the wells by flooding 2 mL of ice-cold sterile distilled water (dH_2_O). The spore suspension was -treated at 60°C for 20 min to kill any vegetative cells and washed 5 times in sterile dH_2_O by centrifuging at 16,000 x *g* for 5 min. Spore suspensions were visualized under a microscope to ensure 90% sporulation before storage at −20°C.

### Sausage preparation and fermentation

Fermented pork sausages were prepared according to a published protocol [31,32]. Pork (90% lean) was hand mixed with the addition of sterile dH_2_O, salt (2.25%), summer sausage spice mix (Sausage Maker, Buffalo, New York, United States), sodium erythorbate (Sigma-Aldrich, St. Louis, Missouri, United States), curing salt, and dextrose (Sigma-Aldrich). *C. difficile* spores (5 log colony-forming unit [CFU]/g of pork batter) and starter culture *P. acidilactici* at 7 log CFU/g (SAGA™ 200 Kerry Ingredients and Flavours, Rochester, Minnesota, United States) were added to ground pork. The ground pork batter was then hand mixed and minced with a manual grinder (Weston, Strongsville, Ohio, United States) using a 3/8” grinding plate. A sausage formulation, including all ingredients except *P. acidilactici*, was included as an unfermented control. The ground pork mixture was stuffed into synthetic casings (Nojax® Casings 13/16” provided by Viskase, Darien, Illinois, United States) using a jerky gun with a 10 mm stuffing horn (LEM, West Chester, Ohio, United States). Sausages were fermented at 37°C for ~12 h under 85% relative humidity (RH) until pH 5.0 was reached. A Caron humidity-controlled incubator (Caron Products, Marietta, Ohio, United States) was utilized for fermentation to maintain 37°C and 85% RH. The fermented group of sausages was fermented to pH 5.0 and one-half were immediately transferred to a drying oven (VWR, Radnor, Pennsylvania, United States) set to 100°C for cooking to an internal temperature of 66.5°C for ~45 min, where the temperature was continuously monitored using a digital thermocouple (Oakton Instruments, Vernon Hills, Illinois, United States) inserted internally in the sausage. The remaining half of the fermented sausages were uncooked, and all were cooled by transferring to individual gas-impermeable Whirl-Pak™ bags (Nasco, Fort Atkinson, Wisconsin, United States) and submerging in ice-cold sterile dH_2_O and stored at 4°C. The control unfermented group of sausages, without starter culture, was also stored at 37°C for ~12 h under 85% RH, and one-half were subjected to cooking utilizing the method stated above. The remaining half were uncooked and subsequently cooled and stored in Whirl-Pak™ bags at 4°C.

### Bacterial enumeration for sausages

Three sausage samples from each treatment group were analyzed for pH, water activity (a_w_), viable *C. difficile* spores, and *P. acidilactici* populations at 1 h following inoculation and on days 0, 1, 7, 14, 21, 30, 60, and 90 of refrigerated storage. Each sausage was removed from its casing, and both the casing and sausage were together added to a Whirl-Pak™ bag containing 10 mL of phosphate-buffered saline (PBS pH 7.0) and subjected to stomaching for 1 min. After stomaching, 1 mL of the sausage homogenate was pour plated in duplicate with *Clostridium difficile* moxalactam norfloxacin agar supplemented with 0.1% sodium taurocholate (CDMNT). The plates were incubated anaerobically at 37°C for 48 h. In addition, 1 mL of the homogenate was added to 9 mL of CDMNT broth and enriched for 24 h under anaerobic conditions. Following enrichment, a 5 mL aliquot of the broth was subjected to alcohol shock by adding 5 mL of 100% anhydrous ethanol (Sigma) for 1 h to eliminate vegetative bacteria. The broth was subsequently subjected to centrifugation at 4000× *g* for 10 min, and the pellet was resuspended in 0.5 mL of PBS and pour plated with CDMNT agar. The CDMNT agar plates were incubated anaerobically at 37°C for 48 h. Moreover, the population of *P. acidilactici* present in each of three sausage samples at each time point was enumerated by plating on de Man, Rogosa, and Sharpe agar (Oxoid) with incubation at 37°C for 48 h [33].

The pH and a_w_ of the fermented pork summer sausages were measured as described by Hristo *et al*. [34]. Briefly, pH was determined at 25°C by inserting a pre-calibrated pH meter probe (Thermo Fisher Scientific, Waltham, Massachusetts, United States) directly into three sausage samples from each treatment group by time point. For measuring a_w_ at room temperature (25°C)_,_ a calibrated a_w_ meter (Rotronic, Hauppauge, New York, United States) was used, and sausage samples from each treatment group were cut into small pieces and placed in a plastic sample cup. The bottom of the cups was completely covered with sausage sample, placed in the a_w_ meter, and readings were recorded following humidity and temperature stabilization of the instrument.

### Statistical analysis

All experiments included triplicate samples for each treatment, and the study was repeated 3 times. The experiment was a split-plot design with the whole plot as the original ground pork batter, which was split into two groups with or without starter culture (control unfermented and fermented). These two batter groups were further split into two groups of cooked and uncooked (control unfermented cooked and fermented cooked), making this a split-split-split plot with randomized complete block. The treatment groups, control unfermented, fermented, control unfermented cooked, and fermented cooked, are the independent variables and *C. difficile* spore counts (CFU/g) are the dependent variable. The data were analyzed using the PROC-GLIMMIX procedure of SAS version 9.4 (SAS Institute Inc., Cary, North Carolina, United States), and differences between the means were considered significantly different at p<0.05. The fixed effects were the treatment groups and the random effects were considered the treatment group and day of storage interaction. A least significant difference test was used to differentiate the variations (p<0.05) with appropriate corrections for multiple comparisons due to replication, treatment, and time on spore counts.

## Results and Discussion

**Figure-1:**
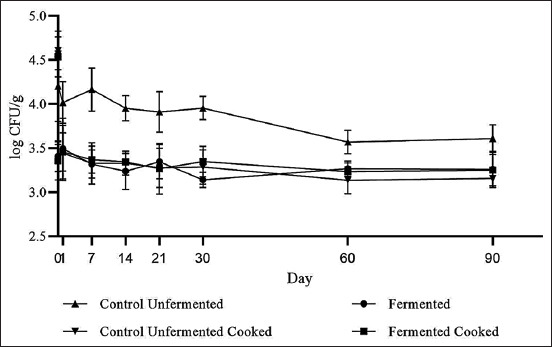
Viability of *Clostridioides difficile* spores in fermented pork sausage.

Results revealed that *C. difficile* spores remained viable under an acidic pH of 5.0 in fermented pork summer sausage during 3 months of refrigerated storage in all treatment groups. *C. difficile* spore viability in control unfermented treatment was significantly different on day 0 from the fermented, fermented cooked, and control unfermented cooked treatments (p<0.05); however, there was no significant difference among the latter three treatment groups throughout 90 days of storage (p>0.05) (Figure-1).

**Figure-2:**
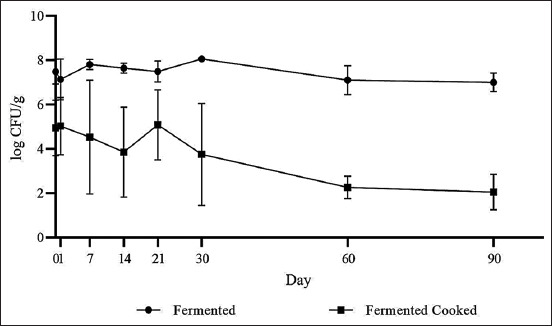
*Pediococcus acidilactici* populations recovered from fermented pork sausage during storage.

On day 90 of storage, the control unfermented sausages yielded ~4.0 log CFU/g of *C. difficile* spores compared to ~3.5 log CFU/g recovered from fermented samples and the control unfermented cooked samples, identifying spore viability in all treatment groups. *P. acidilactici* counts in the fermented group remained at ~7.5 log CFU/g throughout 90 days of storage, but declined in the fermented cooked group from day 21, reaching ~2 log CFU/g by the end of storage (Figure-2).

The pH of control unfermented sausage was 5.68 on day 0 and 5.61 on day 90, with significant variations on days 14 and 60 (Table-1). The pH of control unfermented cooked sausages was 5.73 on day 0, which significantly increased to 6.08 by the end of the storage period (p<0.05), which is consistent with pork characteristics [35]. The pH of the unfermented sausage groups was consistent with agriculture standards for pork meat [36]. On the other hand, the pH of fermented sausages was ~4.8 on day 0, where it increased to 5.04 and 5.31 (p<0.05) on day 90 in uncooked and cooked samples, respectively. The significant increase in pH observed in cooked fermented sausages is consistent with a previous study, where a significant increase in pH of cooked sausages from 4.75 to 4.84 was observed over 8 weeks of storage [37]. Furthermore, no significant difference in a_w_ was observed within the treatment groups during 90 days of storage, with an average of 0.9 for the cooked treatment groups and 0.93 for the uncooked (fermented only) treatment groups (p>0.05) (Table-2).

**Table-1:** Average pH of fermented and unfermented pork sausage with and without cooking.

Day	Control unfermented	Control unfermented cooked	Fermented	Fermented cooked
Day 0	5.68	5.73	4.80	4.81
Day 1	5.69	5.89*	4.92*	5.05*
Day 7	5.73	5.94*	4.74	5.02*
Day 14	5.81*	5.99*	4.65*	5.06*
Day 21	5.61	6.00*	4.72	5.15*
Day 30	5.58	6.10*	4.72	5.19*
Day 60	5.39*	6.19*	4.73	5.32*
Day 90	5.61	6.08*	5.04*	5.31*

* Significant difference between day 0 and sampling day within a treatment group (p<0.05)

**Table-2:** Average water activity of fermented and unfermented pork sausage with and without cooking.

Day	Control unfermented	Control unfermented cooked	Fermented	Fermented cooked
Day 0	0.936	0.910	0.938	0.898
Day 1	0.938	0.941	0.944	0.907
Day 7	0.937	0.898	0.919	0.902
Day 14	0.935	0.908	0.919	0.883
Day 21	0.925	0.901	0.929	0.870
Day 30	0.926	0.900	0.887	0.886
Day 60	0.925	0.912	0.930	0.908
Day 90	0.929	0.905	0.935	0.889

No significant difference within treatment groups (p>0.05)

Hypervirulent *C. difficile* isolates have been detected in live pigs, pork sausage manufacturing plants, and retail pork products [17,27]. The United States Department of Agriculture (USDA) guidelines for meat fermentation are based on literature evaluating pH and temperature parameters for controlling pathogens such as *Escherichia coli* O157:H7 [29]. A pivotal study on the fate of *E. coli* 0157:H7 in fermented summer sausage identified that a 5 log reduction in pathogen counts could be achieved with fermentation to a final pH of 5.0, followed by heating to an internal temperature of 54°C for 30 min, and a 7 log reduction in counts with heating for 60 min at 54°C [28]. However, the results from this study revealed that *C. difficile* remained viable in pork summer sausage despite achieving the aforementioned pH and even higher cooking temperature. This could be attributed to the differences in the heat and acid resistance between the vegetative cells of *E. coli* O157:H7 and *C. difficile* spores. Although the effect of heat and acidity on the viability of *C. difficile* spores in meat has not been investigated, Rodriguez-Palacios *et al*. [38] reported that *C. difficile* vegetative cells survived the USDA recommended meat cooking temperature of 71°C for 2 h in phosphate-buffered saline. In addition, *C. difficile* spores have been found to be more resistant to temperatures between 60°C and 75°C [39]. Thus, *C. difficile* spores are expected to survive in the acidity and cooking commonly employed in fermented pork summer sausage, thereby highlighting the need for effective intervention strategies to reduce spore contamination in RTE meat products. Although, *C. difficile* spores require bile salts such as cholate, taurocholate, and glycocholate to stimulate germination [40], spores would unlikely germinate and enter the vegetative state in a food product. Since *C. difficile* spores were found to survive in pork summer sausage during production and storage, there is a possibility that at-risk individuals could be prone to *C. difficile* infection as they are also prone to other pathogens of concern (*E. coli* 0157:H7, *Salmonella enterica* spp., and *Listeria monocytogenes*) in sausage products noted by FSIS [29].

Many RTE meats, including fermented sausages, which are not heated or heated to a lower internal temperature, contain preservatives to control spoilage bacteria and foodborne pathogens. Sodium nitrite is a preservative commonly used to control *C. botulinum* spores, as well as spoilage bacteria in RTE, cured meat [41,42]. However, a recent study reported that even vegetative cells of *C. difficile* are resistant to the USDA recommended levels of nitrite (200 ppm) and nitrate (500 ppm) in RTE meat products [43]. The study also noted that the use of both preservatives together did not exert a synergistic effect against *C. difficile*. It is highlighted that this study was conducted with vegetative *C. difficile*, and spores being more resistant than vegetative cells to antimicrobials. It is logical to conclude that nitrites and nitrates would not kill *C. difficile* spores if present in RTE meat products. The aforementioned results documenting the resistance of *C. difficile* spores to a commonly used preservative in RTE meat, along with our results showing the survival of *C. difficile* spores under a pH of 5.0 and cooking temperature of 66.5°C for 45 min in fermented sausages, underscore the potential risk of the pathogen in RTE meat.

## Conclusion

Fermentation and cooking (66.5°C for 45 min) of fermented pork summer sausage resulted in only marginal reduction in *C. difficile* spores. The pH of <5, fermentation for 12 h, and subsequent cooking to an internal temperature of up to 66.5°C did not reduce *C. difficile* spores in pork summer sausage stored at refrigeration temperature for 3 months (*p*>0.05). However, follow-up research to determine the outgrowth potential of *C. difficile* spores during temperature-abuse storage and higher cooking temperatures above 75°C during the processing of fermented pork summer sausage is warranted. This study also highlights the need for further research on the potential risk of spore transmission to humans from live pigs, processing facilities, and meat products. As a suggestion for future implementation in meat safety mandates, it is recommended to consider *C. difficile* as a foodborne hazard in minimally cooked and fermented summer sausage products that may potentially contribute to the incidence of community-associated *C. difficile* infections in at-risk and immunocompromised individuals.

## Authors’ Contributions

GF and KV: Conceived the study. GF, HY, CC, and AJP: Produced the fermented pork summer sausages and performed enumeration time points. GF: Analyzed data and drafted the manuscript. KV: Main editor of the manuscript. AJP: Assisted in the manuscript editing. All authors have read and approved the final manuscript.
